# Natural Products Beyond Inhibition: A Mechanistic Framework Spanning Pockets, Interfaces, and Kinetic Barriers

**DOI:** 10.3390/molecules31101577

**Published:** 2026-05-09

**Authors:** Shuo Miao, Huadong Zhao, Aizhe Liu, Ning Xu, Xiangsheng Liu, Xie Wang

**Affiliations:** 1 College of Pharmaceutical Sciences, Zhejiang University of Technology, Hangzhou 310014, China; 2 The Key Laboratory of Zhejiang Province for Basic and Clinical Application of Functional Nucleic Acids, Hangzhou Institute of Medicine (HIM), Chinese Academy of Sciences, Hangzhou 310022, China; 3 State Key Laboratory for Quality Ensurance and Sustainable Use of Dao-Di Herbs, Beijing 100700, China

**Keywords:** natural products, mechanism of action, conformational regulation, biomolecular interfaces, kinetic trapping

## Abstract

Natural products display exceptional chemical diversity and a broad range of mechanisms of action that are not adequately captured by traditional classifications based on target class, pharmacological phenotype, or chemical scaffold. Such classification schemes often lead to fragmented understanding of mechanisms of action, obscuring the unified principles underlying different target systems while failing to recognize the stage-dependent mechanisms exhibited by the same molecule in varying contexts. Here, we propose a unified “space–interface–time” framework to classify the mechanisms of action by examining the physical principles through which natural products reshape the functions of different biomolecules. Within this framework for unifying the classification of natural product mechanisms of action, geometry-driven binding site occupancy and conformational constraints are assigned to the spatial dimension; induction or stabilization of multicomponent complexes and kinetic regulation of state lifetimes are assigned to the interfacial and temporal dimensions, respectively. Finally, we discuss the conceptual and technical challenges of bridging static structural snapshots with dynamic in vivo pharmacology, and highlight emerging opportunities offered by time-resolved structural methods and the integration of molecular dynamics, machine learning, and biophysical workflows for mechanism-guided drug discovery.

## 1. Introduction

Natural products exhibit exceptional structural diversity and correspondingly diverse mechanisms of action [1,2,3]. Although traditional classification schemes based on target class, pharmacological phenotype, or chemical scaffold are practical, they often group natural products with distinct physical regulatory mechanisms under the same label [3,4]. A single natural product may exhibit classic inhibitory effects under certain conditions, yet exert its pharmacological effects through complex interface interactions in other contexts [1,5,6]. FK506 is an example of this, as it binds to FKBP12 with high affinity and can inhibit FKBP12 peptidyl-prolyl cis-trans isomerase (PPIase) activity. However, its immunosuppressive effects primarily arise from the surface structure of the FK506-FKBP12 complex, which blocks NFAT-dependent T-cell activation by binding to and inhibiting calcineurin. These differences profoundly influence the drugability, selectivity, and signaling outcomes of natural products, yet they are often obscured by classification approaches centered on targets or phenotypes. More broadly, structurally similar natural products may exhibit distinct mechanisms of action, while structurally unrelated compounds may share identical regulatory principles [3,7].

Although recent reviews have categorized natural products as sources of drug leads [3,4], and increasing studies have incorporated selected natural products within molecular-glue paradigms [1,5,6], few attempts have been made to integrate protein, nucleic acid, and flexible ligand frameworks within a single framework. The absence of such a unified framework results in significant gaps in mechanistic understanding. When reviewing natural product classes that share convergent mechanisms of action (such as molecular glues), focusing solely on a single dimension (target, phenotype, or chemical scaffold) inevitably leads to fragmented descriptions of mechanisms and obscures the unified mechanistic principles across different target systems. Furthermore, existing studies on the mechanisms of natural products rarely discuss the time-dependent characteristics of natural products and their transient intermediates, despite residence time and binding kinetics being widely recognized as core determinants of in vivo pharmacological actions [8,9,10]. Most critically, existing reviews lack descriptions of how a single natural product molecule exhibits distinct mechanisms of action across different stages of its effects. This multidimensional overlapping characteristic, as exemplified by FK506 and brefeldin A, is precisely what current classification systems fail to adequately capture.

The limitations of traditional frameworks also manifest in their reliance on a static perspective of biological targets. In fact, the function of biomolecules is governed by their conformational energy landscapes and transitions between states. The binding accessibility, modular interfaces, and state lifetimes of target molecules directly influence the pharmacological output of natural products [2,8,9,10,11,12,13]. Leveraging the characteristics of dynamic conformational changes in biomolecules, natural compounds, with their rigid frameworks and multi-point interaction capabilities [3,14], can effectively shift conformational distributions, remodel multi-component complexes, or capture transient intermediates, thereby yielding pharmacologically exploitable conformations [1,5,6,15].

To address these limitations, we propose a unified “Space–Interface–Time” (SIT) framework for classifying natural product mechanisms according to the physical principles through which they reshape biomolecular function. Rather than replacing target-, phenotype-, or structure-based paradigms, the SIT framework is intended as a complementary, mechanism-centered perspective for comparing natural products across different biomolecular systems. To clarify its conceptual position relative to these classical paradigms, a concise comparison is provided in Table 1.

In this framework, ‘Space’ refers to geometry-driven complementarity and occupancy that shifts conformational ensembles, ‘Interface’ refers to induced or stabilized intermolecular contacts that define activity at the level of multicomponent complexes, and ‘Time’ refers to kinetic modulation that prolongs state lifetimes and enables the trapping of transient intermediates [6,8,9,10,15,16]. We then apply this framework to diverse natural products and target systems, using structural and mechanistic evidence. Our aim is to provide a cross-system language for mechanism-guided discovery and design.

## 2. Space-Based Strategies

To illustrate how the Space–Interface–Time (SIT) framework can be applied across structurally diverse natural products and distinct biomolecular systems, selected representative examples are summarized in Table 2. Rather than serving as an exhaustive inventory, this overview highlights instructive cases under the three primary dimensions of the framework together with the corresponding biomolecular systems, key lines of supporting evidence, and mechanistic outcomes.

As outlined in Table 2, natural products can reshape biomolecular function through three recurrent physical dimensions: spatial constraint, interfacial modulation, and time-dependent control. We begin with the spatial dimension, in which pharmacological activity arises from the ability of natural products to impose conformational constraints on specific molecular systems. Natural products may achieve this either by occupying defined spaces within targets and thereby restricting their conformational freedom, or by serving as rigid scaffolds that preorganize flexible ligands into bioactive conformations [7,17,18]. The following cases illustrate how this principle is realized in multiple forms across different spatial contexts.

**Figure 1:**
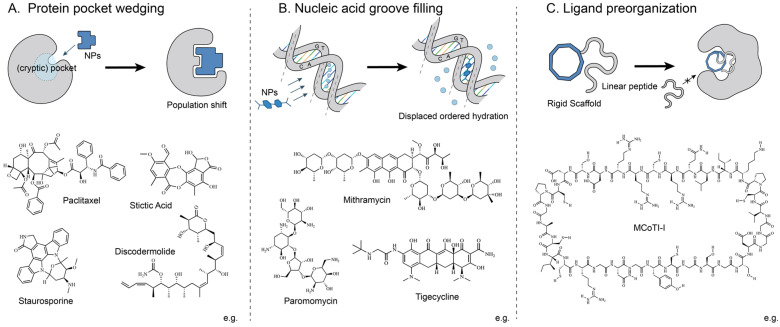
Representative modes of space-driven action by natural products. (**A**) Protein pocket wedging: natural products enter binding pockets (cryptic pockets) in proteins. (**B**) Nucleic acid groove filling: natural products insert into grooves of DNA or RNA. (**C**) Ligand preorganization: natural products act as rigid scaffolds to spatially constrain flexible ligands.

In the context of protein systems (Figure 1A), natural products have been observed to enter binding pockets or transiently accessible cryptic cavities, thereby effecting a reshaping of the local geometry through shape complementarity and induced-fit effects. This can trigger both local and even global conformational changes via allosteric coupling.

Within nucleic acid systems, natural products are known to insert into the minor grooves of DNA or the deep grooves of RNA (Figure 1B). During this process, the natural product displaces the ordered hydration layer within the groove and forms a dense non-covalent contact network. This constrains the dynamic conformational ensemble of nucleic acids in their functional state.

In the context of flexible ligand systems (Figure 1C), the rigid scaffold of natural products serves as a conformational preorganisation platform. Following the grafting of flexible linear peptide epitopes onto these natural product-derived rigid scaffolds, a substantial narrowing of the conformational space of the epitopes is observed, and correspondingly, a reduction in the entropic penalty of binding.

Despite the diversity of molecular targets, these modes of action have been shown to converge on a shared physical outcome: occupation of a defined space by a rigid molecule induces a population shift in the dynamic conformational ensemble towards stabilized functional conformational states [97,98,99].

### 2.1. Protein Pocket Wedging by Natural Products

Many natural products, due to their great molecular rigidity and high structural complexity [14], can occupy specific cavities [18,100] or transiently accessible cryptic pockets [101,102,103,104] within target proteins. In a mode of action analogous to protein wedging [105], natural products not only fill binding pockets through shape complementarity and van der Waals interactions but also elicit induced-fit effects [106]. This leads to local spatial readjustments, manifested as binding-site volume expansion or entrance-gating rearrangements, among others. These changes further influence neighboring structural elements (such as loop regions, helices, and loop-gating residues) through allosteric coupling [107], triggering local or global conformational changes [11,108,109] and altering protein biological activity.

From the perspective of the energy landscape [13,110], this process exerts both thermodynamic and kinetic effects on protein conformational ensembles [11,12,111,112]. (1) Conformations consistent with the binding of the natural product become thermodynamically stabilized [12]. (2) The energy barriers for conformational transitions may increase in certain cases [9]. This enables the protein to adopt a persistent functional state that is less susceptible to perturbation [113]. The following section includes representative examples featuring target types and binding pocket types, including cytoskeletal elements such as tubulin, signaling kinases such as Lck kinase, the molecular chaperone Hsp90, and the tumor suppressor p53. These cases demonstrate how structurally diverse natural products utilize protein pockets to achieve their pharmacological activities, thereby illustrating the overall utility of this binding mode for a variety of protein targets.

#### 2.1.1. Paclitaxel

The binding of paclitaxel to β-tubulin serves as a classic example of a molecular wedge. The ligand occupies the paclitaxel binding site, influencing tubulin conformation through site-specific volume expansion, which is reflected in the local geometry surrounding the binding site [17]. Cryo-EM studies demonstrate that upon Taxol binding, the volume of the pocket on β-tubulin increases from 883 Å^3^ to 996 Å^3^ [18]. At the contact level, the drug tightly packs within the binding pocket, making shape complementarity and van der Waals stacking the most plausible mechanisms describing this interaction. Consequently, paclitaxel shifts fragile GDP microtubules toward the more stable GTP conformation and counteracts hydrolysis-associated rearrangement processes [18,19]. From a functional standpoint, the natural product paclitaxel acts as a microtubule stabilizer, enhancing longitudinal intermolecular interactions among adjacent tubulin heterodimers to ultimately drive its antineoplastic efficacy [18,20].

#### 2.1.2. (+)-Discodermolide

(+)-Discodermolide (DDM) is a potent microtubule-stabilizing agent and has been reported to act synergistically with paclitaxel in cellular and in vivo models. In the X-ray tubulin-DDM complex, DDM is deeply buried in the β-tubulin taxane pocket with a hairpin-like conformation. Compared to paclitaxel, DDM completely fills the cavity formed by the H6/H7 helices, the H6-H7 loop, and the N-terminal region of the M-loop [21]. This space-filling fit provides a structural rationale that is consistent with DDM’s ability to promote a microtubule-stabilizing tubulin conformation [21]. Similarly, by promoting microtubule polymerization, DDM retains cytotoxic activity in paclitaxel-resistant tumor cell lines and can further potentiate the efficacy of paclitaxel in ovarian cancer models [21,22].

#### 2.1.3. Staurosporine

Staurosporine is a broad-spectrum kinase inhibitor with nanomolar potency [23]. Its rigid and fused carbazole core fits tightly into the conserved ATP-binding cleft. Authors demonstrated that staurosporine occupies the ATP site in the Lck kinase domain, which is a Src-family tyrosine kinase that participates in early T-cell receptor signaling. Its dysregulation has been implicated in oncogenic and proliferative signalling in multiple hematologic and solid cancers [23,24]. Upon binding, staurosporine induces a conformational shift in the glycine-rich loop, most evident in the β1 strand, which moves toward the ligand (~1.7 Å) and increases packing contacts [25].

#### 2.1.4. Geldanamycin

Geldanamycin binds within a distinct and highly conserved pocket (approximately 15 Å deep) in the N-terminus of the geldanamycin-binding domain of Hsp90 [26]. Within this complex, the ligand adopts a compact, polypeptide-like conformation exhibiting extensive surface complementarity that supports dense van der Waals interactions. Given the similarity between the pocket and substrate-binding sites, along with the conformational gating at its entrance, geldanamycin can be regarded as a deep-pocket occupant. It disrupts Hsp90-mediated conformational maturation/refolding and promotes degradation of client substrates [26]. Specifically, this N-terminal ATP-competitive blockade deprives numerous oncogenic client proteins of their required chaperone stabilization, ultimately disrupting multiple oncogenic pathways [27].

#### 2.1.5. Stictic Acid

Rigid natural product scaffolds can exploit cryptic pockets that are only transiently accessible. In the stictic acid–p53 system, the ligand enters the transiently open L1/S3 pocket, and subsequent induced-fit adjustments improve shape complementarity within the site [28]. The rotamer state of Leu114 is crucial for opening a binding site large enough to accommodate stictic acid, as Leu114 plays a key role in the opening process of the L1/S3 pocket. Stictic acid may make more interactions within the L1/S3 pocket, which could give it a greater capacity to stabilize the p53 core domain, rather than reflecting nonspecific surface association [28]. Upon binding to the L1/S3 pocket, small molecules such as stictic acid can induce mutant p53 proteins to refold into a wild-type-like conformation. At the biological level, stictic acid supports the concept that engagement of the L1/S3 pocket can restore p53-dependent transcriptional output and cancer-suppressive potential [29].

### 2.2. Nucleic Acid Groove Filling by Natural Products

Similar to protein wedging, natural products can fit into the nucleic acid’s groove [114,115,116], primarily by adapting to the groove geometry and forming non-covalent interaction networks. Natural products with rigid structures can insert into the minor groove of B-form duplex DNA or into deep grooves of RNA, through geometric complementarity and electrostatic interactions [117]. During this process, the natural product ligand displaces the ordered hydration network within the groove [118,119], which can contribute a favorable thermodynamic driving force [120,121]. At the same time, directed hydrogen bonds, van der Waals contacts, electrostatic attractions, CH–π interactions, and π-stacking collectively build up a network of non-covalent interactions [116]. In certain cases, natural products can also form more rigid dimeric complexes by metal coordination [122], thereby utilizing water-mediated hydrogen bonding to lock key nucleotides into specific functional conformations [38,122,123,124,125].

Natural products alter groove width and induce conformational fitting through these interactions [30,33,126], enabling conformational rearrangement within the groove region without significant changes to nucleic acid structural parameters [30,127,128]. Under a combination of these multiple non-covalent forces, the shape and conformation of the nucleic acid groove are confined to a restricted subset of conformations [129], such that the dynamic conformational ensemble of the nucleic acid in solution [130,131] is significantly biased toward and ultimately stabilized in functional conformational states [12,132].

The following representative examples will illustrate from three perspectives: stabilization of groove-recognition geometry, suppression of base flipping, and locking of the conformational switch associated with ribosomal A-site decoding. How the volume occupation and conformational restriction imposed by natural products translate local structural perturbations of nucleic acids into appreciable biological functional consequences.

#### 2.2.1. Netropsin

Like protein wedges, the natural product Netropsin can also function as a shape-matching wedge within the DNA groove. The netropsin molecule is twisted in a screw sense that favors insertion into the minor groove of B-DNA and binds preferentially to clusters of A/T base pairs [30]. Netropsin displaces the hydration spine from the minor groove of B-DNA, a process that provides an entropic contribution to DNA-drug binding [31]. The NH groups of netropsin form bifurcated (three-center) hydrogen bonds to adenine N3 and thymine O2 on adjacent base pairs, while the van der Waals interaction involving adenine C2 hydrogens is the primary source of sequence specificity. Following binding, although the minor groove was locally forced open by 0.5–2.0 Å, no significant systematic changes in helical rotation or base stacking were observed [30]. Netropsin has been reported to possess antiviral activity in earlier literature. By selectively occupying the AT-rich sequences within viral replication origins, the netropsin molecules act as physical roadblocks that prevent viral proteins from unwinding the viral DNA, thereby potently inhibiting the replication of viruses [32].

#### 2.2.2. Spirocyclopropylcyclohexadienone

Similarly, Spirocyclopropylcyclohexadienone (SCPCHD) can also utilize this groove-filling strategy to stabilize DNA double strands. When bound to AT-rich regions, the hydrophobic surface is sandwiched between the backbones of the two DNA strands. This arrangement forms an extended noncovalent CH–π interaction network. Through this CH–π interaction network, the SCPCHD adduct induces conformational fitting within the minor groove, thereby preventing severe distortion of the B-DNA conformation [33]. At this point, SCPCHD-modified DNA becomes a substrate difficult for downstream repair enzymes to process, effectively preventing base flipping in DNA. As a result, replication, transcription, and other nucleic acid metabolic processes are inhibited, leading to high cytotoxicity toward tumor cells [33,34].

#### 2.2.3. Mithramycin

Unlike the AT binding, the potent antagonistic activity of the natural product mithramycin (MTM) against the oncogenic transcription factor EWS–FLI1 is mediated by the insertion of a metal-coordinated dimer into the DNA minor groove [35]. The two MTM monomers form a nearly ideal octahedral coordination sphere with Zn^2+^ and two water molecules. This rigid core structure wedges itself into the minor groove of DNA (groove width ~14 Å) [36]. MTM and its derivatives interfere with FLI1 in different states. At sub-micromolar concentrations, they can form FLI1-DNA-MTM ternary complexes. However, at higher concentrations or when MTM binds to alternative DNA registers that sterically overlap with the FLI1 binding site, steric clashes occur. Consequently, the stabilizing effect diminishes, and MTM acts as a competitive antagonist [35,36].

#### 2.2.4. Paromomycin

Paromomycin, an aminoglycoside antibiotic, disrupts translation accuracy by binding to the tRNA decoding A-site of bacterial 16S rRNA, and retains antiribosomal and antibacterial activity [37,38]. In the 2.5 Å crystal structure of an 18 bp RNA double helix that contains two minimal A-site motifs, two paromomycin molecules bind independently to the two sites [38]. Each ligand is accommodated within an enlarged deep groove created by the A-site, where it is anchored by a dense network of direct and water-mediated hydrogen bonds (13 direct H-bonds and 8 waters mediate 12 additional contacts). Importantly, paromomycin binding promotes and stabilizes the bulging of A1492/A1493, and this stabilization biases the A site toward a decoding-like conformational switch [38].

#### 2.2.5. Tigecycline

At 3.3 Å resolution, X-ray crystallography of the Thermus thermophilus 70S ribosome initiation complex revealed that tigecycline binds at the primary tetracycline site formed by h31/h34 of 16S rRNA on the 30S head [39]. Tigecycline exerts its antibacterial effect during the decoding process by disrupting the recognition step of the initial codon by tRNA [39,40]. The characteristic structure of tigecycline is the 9-tert-butylglycylamido moiety and its participation in stacking interactions with the π-system of C1054. Strong electron density for the moiety of tigecycline indicates that it adopts a very rigid conformation, and this rigidity may facilitate its stacking interaction with C1054. Overlay analysis with the ribosome structure bound to TetM reveals overlap between the 9-substituent and TetM domain, providing a structural basis for tigecycline’s resistance to TetM-mediated rescue [39].

### 2.3. Ligand Preorganization by Natural Products

In the previous sections on proteins and nucleic acids, natural products were shown to remodel local pocket/groove geometry via their rigid structures, thereby reshaping or stabilizing specific conformational states of the target [133,134]. The conformational restriction strategy based on geometric constraints is not only applicable to the target molecules themselves but also serves as a powerful method for optimizing flexible ligands [135]. Molecular grafting [136,137] has emerged as an effective method that leverages natural products to impose geometric constraints, thereby preserving the functional conformation of the flexible ligand.

This strategy employs natural product-derived cyclic peptides or knottin structures as highly rigid molecular scaffolds. Then the flexible ligands, such as linear peptide epitopes, are precisely grafted onto specific sites of the scaffolds. This method preorganized the unstructured linear fragment into a specific bioactive conformation, effectively mitigating the significant entropic penalty incurred when flexible ligands bind to target molecules [138]. Consequently, the geometric modulation of natural products extends beyond merely wedging into target pockets or grooves, encompassing the conformational preorganization of ligands as well. Both mechanisms aim to restrict the accessible conformational ensemble and stabilize functional conformation states.

The following text will discuss how flexible ligands exhibit improved pharmacologically relevant properties following engineering modifications, utilizing the natural cyclotide Kalata B1 and Momordica cochinchinensis trypsin inhibitor-I (MCoTI-I), as well as the agouti-related protein (AgRP), as representative cases.

#### 2.3.1. Kalata B1

The application of short peptide epitopes has been limited by their poor bioavailability and stability under physiological conditions. The inherent structural flexibility makes them more susceptible to enzymatic cleavage. Furthermore, the loose structure of peptides in solution results in a low proportion of biologically active conformations within their conformational ensemble [41]. In this study, the authors grafted the peptide epitope involved in VEGF-A antagonism onto the highly stable Kalata B1 cyclopeptide backbone. This modification enhanced in vitro stability without perturbing the overall folding structure, while also preserving full biological activity in the antiangiogenesis cell-based assay. Despite the grafted loop-3 region of cpr3 exhibiting a disordered and flexible segment, it showed significant inhibitory activity with an IC_50_ of 12 µM. In contrast, the linear poly-R epitope and other grafts were significantly less active [42]. Consistent with this rationale, Kalata B1-based grafted constructs have shown antiangiogenic activity comparable to that of the parent linear epitope and have been discussed as antiangiogenic and anticancer leads [43].

#### 2.3.2. Momordica Cochinchinensis Trypsin Inhibitor-I

Based on the rigid molecular platform of Momordica cochinchinensis trypsin inhibitor-I (MCoTI-I), the authors grafted the α-helical peptide PMI derived from p53 onto loop 6 of the cyclotide. The *N*-terminus of PMI was fused to a linker peptide derived from apamin, and the modified apamin-PMI hybrid peptide was grafted between Ser31 and Gly33 [44]. The resulting grafted cyclic peptide was named MCo-PMI. Cell experiments confirmed that the cyclotide MCo-PMI can also target intracellular Hdm2 and HdmX, highlighting its specificity in regulating the p53 signaling pathway in vitro. The chemical shift changes were concentrated around loop 6, while the backbone amide proton shifts from loops 1–5 are well within a 0.2 ppm range, indicating only minor changes. This suggests that the cyclotide folding structure in MCo-PMI is mostly preserved [44].

#### 2.3.3. Agouti-Related Peptide

The 34-residue, cystine-knot C-terminal domain of Agouti-related peptide (AgRP) provides a compact, disulfide-stabilized scaffold. In this framework, the native loop 4 segment was replaced with an RGD-bearing loop and diversified by combinatorial randomization of residues flanking the RGD sequence. In fact, although the RGD sequence can be promiscuously recognized by multiple integrins, its biological activity is highly dependent on its own sequence and structural environment. Due to the lack of conformation specificity conferred by folded protein domains, the affinity of linear RGD peptide sequences is significantly reduced [45]. Therefore, this study utilized macromolecular modeling to determine the size of this loop and the register of the RGD motif, thereby reproducing the optimal binding conformation on a rigid scaffold [46].

## 3. Interface-Based Strategies

For a long time, natural products have been widely recognized for their ability to occupy the binding site of a single target molecule. Over the past decades, with the continued advances in structural biology and chemical biology, natural products have increasingly highlighted an equally powerful yet mechanistically more distinctive paradigm—modulation of biomolecular interfaces.

Beyond occupying the binding site of a single target, natural products leverage their unique structural and physicochemical properties to localize at composite interfaces formed by two or more components [6,138], such as protein–protein, protein–RNA, and RNA–RNA interfaces. In certain systems, they may also act upon intramolecular interfaces formed by the folding of a single macromolecule [139]. Natural products can regulate interactions through multiple pathways. They can fill interfacial cavities [50], bridge both binding partners across the interface [73], or remodel protein binding surfaces to form a neomorphic interface [56]. These processes ultimately alter the binding equilibria and kinetic barriers of macromolecular interactions [140,141].

This suggests that the understanding of the pharmacological mechanisms of natural products is evolving: biological effects are not solely determined by the properties of any single component, but are more closely related to the multicomponent complexes induced or stabilized by natural products.

### 3.1. Protein Interfacial Assembly by Natural Products

**Figure 2:**
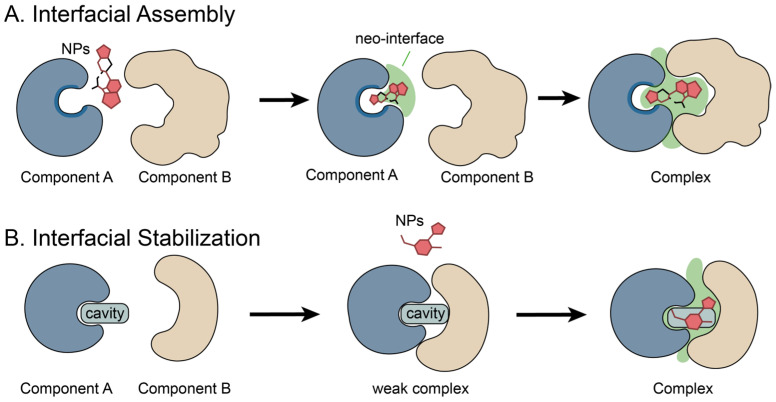
Representative modes of protein interface-driven action by natural products. (**A**) Interfacial Assembly: Natural products promote the association of proteins that exhibit non-existent or only weak prior interactions. (**B**) Interfacial Stabilization: Natural products stabilize pre-existing interfaces (cavities) at protein–protein junctions.

Natural products and their derivatives, in addition to exerting steric blockade by occupying the binding site of a single target molecule, can also act as molecular glues to fill or remodel protein–protein interaction interfaces (Figure 2A) [6]. They can bring two proteins into proximity and induce/enhance a composite interface jointly formed by the ligand and the proteins [16,48,142], thereby compensating for the original weak affinity and/or interaction between the proteins. In this process, natural products rely on the preorganized conformations and shape complementarity of their scaffolds to localize at the interprotein junction or at the bottom of the receptor pocket, and to participate in contacts on both sides within the complex.

By filling interprotein cavities [48], extending the substrate-binding pockets [50], or chemically remodeling the interface to generate neomorphic contacts [56], they promote the assembly and stabilization of ternary complexes. In these ternary complexes, direct protein–protein contacts are often relatively sparse, and the natural product is required as a scaffold to bridge the binding interface. This further supports that activity is not determined by any single component, but rather by the ternary complex formed by the ligand and the proteins themselves.

To better describe this interface-remodeling mechanism, the following text will present four examples of natural products and natural product-inspired molecular glues and introduce their diverse modes of action.

#### 3.1.1. Rapamycin

As a macrolide natural product with significant immunosuppressive activity, rapamycin promotes the formation of the FKBP12-rapamycin-FRAP (FRB) ternary complex by acting as a molecular glue. The 2.7 Å crystal structure reveals that the conformationally constrained triene moiety pre-organizes the macrocyclic skeleton, exposing the rapamycin-binding surface as part of the complex surface to the FRB domain upon FKBP12 association [47]. In this structure, rapamycin is largely confined to the interprotein junction, forming the major portion of the binding interface, while direct contact between FKBP12 and FRB is sparse.

This mechanism of action of rapamycin exemplifies how natural products mediate interactions through interfaces formed by ligands and proteins. Through the stereochemically constrained macrocyclic skeleton, rapamycin induces protein association, with the resulting protein-ligand complex (not individual components) serving as the active entity [48].

Functionally, this ternary complex constitutes the structural basis by which rapamycin inhibits mTORC1 signaling, thereby linking rapamycin-induced protein association to geroprotective and anti-aging effects [49].

#### 3.1.2. Indole-3-Acetic Acid

The major natural auxin, indole-3-acetic acid (IAA), enhances TIR1’s substrate-binding activity by acting as a molecular glue to fill the cavity between the two proteins of TIR1 and Aux/IAA, thereby extending the protein-interaction interface. Upon perception by TIR1, auxin modulates gene expression by facilitating the SCF-mediated degradation of Aux/IAA transcriptional repressors. In this manner, co-receptor assembly is converted into transcriptional output, thereby driving auxin-responsive developmental programs [50,51]. The single top surface pocket within the TIR1 leucine-rich repeat (LRR) domain recognizes auxin and its Aux/IAA polypeptide substrates. This pocket is open at the top and accessible only from above. Auxin is anchored at the bottom of the pocket; immediately above it, the IAA7 peptide docks into the upper region of the auxin-bound TIR1 pocket [50]. Structurally, within the ternary complex, the Aux/IAA substrate peptide fills the remainder of the TIR1 binding pocket, effectively sequestering the hormone within its binding site. This leads to the hormone remaining trapped in the pocket until the substrate polypeptide is released [50]. Although IAA has not yet become a mature therapeutic agent, its mechanism of action has clear implications for drug development. IAA itself is being explored in the field of photodynamic therapy for facial seborrhoeic dermatitis and in enzyme-activated prodrug strategies for cancer treatment, highlighting its translational medical value beyond plant biology [50,52].

#### 3.1.3. (3R,7S)-Jasmonoyl-L-Isoleucine

Bioactive jasmonate signalling is mediated by the highly active conjugate (3R,7S)-jasmonoyl-L-isoleucine (JA-Ile), which is functionally and structurally mimicked by the phytotoxin coronatine. The F-box protein Coronatine Insensitive 1 (COI1) mediates jasmonate signalling by promoting hormone-dependent ubiquitylation and degradation of Jasmonate Zim Domain (JAZ) transcriptional repressors, which have been identified as SCFCOI1 substrate targets. In line with this, JAZ proteins associate with COI1 in a hormone-dependent manner, consistent with the view that the jasmonate receptor is a COI1-JAZ complex [53,54]. Hormone recognition requires a JAZ degron, which comprises a COI1-docking α-helix and an adjacent loop region [53,55]. The degron’s amphiphilic helix binds tightly to the upper face of the LRR domain of COI1, positioned next to the ligand site. In the COI1-JA-JAZ complex, JA-Ile/coronatine occupies the bottom of the COI1 pocket, while the *N*-terminal seven residues of JAZ span the pocket entrance as a clamp. It simultaneously established contact with both COI1 and the ligand, thereby effectively sequestering the signaling molecule. Overall, JA-Ile/coronatine makes direct contacts with both COI1 and the JAZ protein, thereby stabilizing the assembled COI1-JAZ co-receptor surface. This structural arrangement is further stabilized by an adjacent inositol pentakisphosphate (InsP5) cofactor [53].

#### 3.1.4. Sanglifehrin A

Schulze et al. described a Sanglifehrin A-inspired tricomplex inhibitor. Small molecules (such as RMC-4998) chemically remodel the surface of Cyclophilin A (CYPA) to form a high-affinity, novel protein–protein interface for the active KRAS G12C. Neither CYPA nor RMC-4998 alone significantly affects KRAS. Instead, all three components are essential for tricomplex formation.

In the CYPA/RMC-4998/KRAS G12C tricomplex, stability is supported by three interactions: (i) direct noncovalent ligand contact between RMC-4998 and the KRAS switch I/II regions; (ii) neomorphic contacts, including hydrogen bonds between specific CYPA residues and KRAS switch I/II residues; and (iii) covalent modification occurring between KRAS and RMC-4998. The assembled tricomplex demonstrated that CYPA blocks the binding interface of KRAS G12C effectors, including CRAF, PI3K, and SOS1 [56,57]. Consequently, tricomplex formation induces the concentration-dependent dissociation of effectors in biochemical assays. This interface mechanism has been demonstrated to promote tumor regression in multiple human cancer models, effectively inactivating oncogenic signaling pathways [56].

### 3.2. Protein Interfacial Stabilization by Natural Products

Natural products also exhibit another structurally selective mode of interfacial modulation when modulating biomolecular interactions. For naturally occurring but thermodynamically or kinetically unstable protein–protein interfaces, natural products can act as interface-stabilizing ligands by selectively binding to interface-associated sites (Figure 2B) [143]. They stabilize the native interface or capture short-lived intermediate complexes by strengthening non-covalent contacts across the interface [15,58].

In this model, natural products do not establish new interaction networks but instead exploit their shape complementarity to insert into pre-existing interfacial cavities (including transient pockets) [144,145]. By filling the cavities, increasing the number of interfacial contacts, and biasing the conformational equilibria [12], they prolong the lifetime of the complex and raise the kinetic barrier to dissociation, thereby achieving interfacial stabilization.

This mechanism is illustrated through two case studies in the following text. Research demonstrates that resveratrol stabilizes the native transthyretin (TTR) tetramer by binding to the T4-binding pocket. Brefeldin A, meanwhile, has been shown to bind to and stabilize the transitory Sec7d-ARF1-GDP intermediate complex.

#### 3.2.1. Resveratrol

Resveratrol and its derivatives (such as piceatannol) effectively inhibit amyloid fibrillation. This process is accomplished by resveratrol binding to the T4-binding site, thereby stabilizing the transthyretin (TTR) tetrameric structure. The T4-binding site constitutes a T4-binding pocket formed by two subunits through twofold rotational symmetry. The binding process of resveratrol and its derived TTR ligands has been demonstrated to be enthalpy-driven. As demonstrated by isothermal titration calorimetry (ITC) and high-resolution X-ray crystallography, this favorable binding enthalpy (ΔH = −15 kcal/mol) is primarily driven by hydrogen bonding with Ser117 and further enhanced by CH···π interactions involving Leu110 [58]. Unlike compounds such as auxin that induce novel protein–protein interactions, resveratrol and its derivatives stabilize the naturally occurring tetrameric interface by filling cavities at the subunit interface, thereby preventing dissociation. Thus, ligand binding at the T4 site represents not just pocket occupation in isolated monomers, but an excellent example of stabilizing the protein interface in the native state [58]. Functionally, stabilization of the native TTR tetramer at the T4 site is associated with anti-amyloidogenic and neuroprotective effects in Alzheimer’s disease [59].

#### 3.2.2. Brefeldin A

The fungal metabolite Brefeldin A (BFA) can block ADP-Ribosylation Factor 1 (ARF1) activation by inhibiting a Golgi-associated Sec7-family guanine nucleotide exchange factor (GEF). The 2.4 Å crystal structure revealed that, rather than blocking the interaction between Sec7 and ARF1, BFA binds to the ARF1•GDP–Sec7 ternary complex [15]. By inhibiting Sec7’s GEF activity as an early intermediate, it traps ARF1 in an unproductive early conformation state (ARF1·GDP). Consistent with this unusual mode of action, only the ternary complex is targeted, with no binding observed to the Sec7 domain or ARF1•GDP proteins. The BFA binding site is located at the protein–protein interface between ARF1 and Sec7. This newly created pocket at the ternary complex interface is essentially a hydrophobic cavity formed by both components. BFA prolongs the lifetime of typically transient intermediates, thereby converting transient intermediates into stable and observable ARF1•GDP–Sec7-BFA complexes [15,60]. This mechanism may underlie host-directed antiviral effects, as BFA disrupts cholesterol trafficking to the plasma membrane and virion transport from early to late endosomes [61].

#### 3.2.3. Fusicoccin A

Fusicoccin A is a canonical exemplar of an interface-stabilization strategy. Its key mode of action is not inhibition of a pre-existing active site on a monomeric protein. Rather, it selectively binds a preassembled 14-3-3–target complex, stabilizes that composite assembly, and thereby achieves functional regulation. In the plasma-membrane H^+^-ATPase system, 14-3-3 proteins recognize the C-terminal mode III phosphorylation motif (YpTV-COOH) and promote pump activation. This interaction is strongly potentiated by fusicoccin. Structural studies show that FC inserts into the conserved amphipathic cavity of 14-3-3 and makes molecular contacts also with the C-terminal end of the peptide [62]. Together, these interactions stabilize the composite interface and promote mutual stabilization of both ligands. Quantitatively, ITC indicates that in the presence of 100 μM FC, the dissociation constant (K_D_) for binding between the H^+^-ATPase mode III phosphopeptide and 14-3-3ζ decreases from 0.09 μM to 0.01 μM (≈9-fold) [62]. The thermodynamic profiles of the aforementioned binding events indicate that the FC effect is considerably different in the various peptides assayed. For some substrates, the increased interfacial contact area yields a more favorable enthalpic contribution. For others, FC-driven desolvation and burial of the hydrophobic surface lead to a more entropy-driven enhancement. Conversely, bulky hydrophobic residues could be disfavored in the presence of FC, due to the steric hindrance of the latter inside the cleft. This behavior is consistent with a pocket-occupancy-driven mechanism that can selectively stabilize or destabilize specific 14-3-3 complexes [62]. In planta, FC-A enhances plant growth by stabilizing the protein–protein interaction between plasma membrane (PM) H^+^-ATPase and 14-3-3 in guard cells [63].

### 3.3. Nucleic Acid Interfacial Modulation by Natural Products

**Figure 3:**
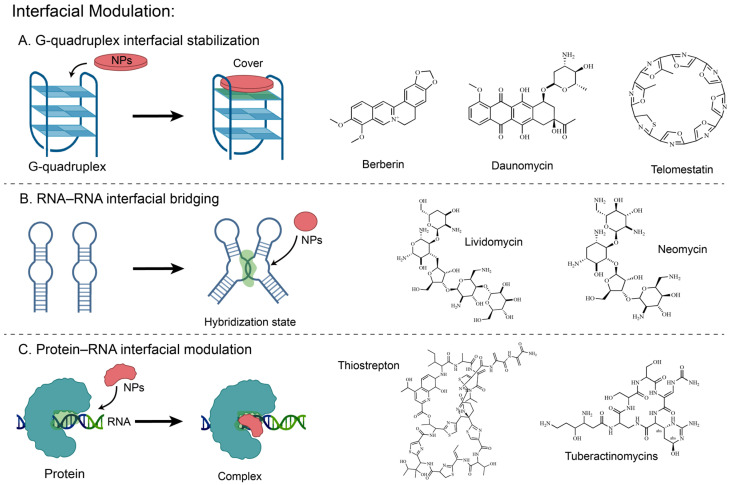
Representative modes of nucleic acid interfacial modulation by natural products. (**A**) G-quadruplex interfacial stabilization: natural products bind to the external G-tetrad surfaces of G-quadruplexes, primarily through end-stacking and groove-binding interactions. (**B**) RNA–RNA interfacial bridging: Natural products engage composite RNA–RNA junctions by simultaneously contacting both strands, acting as intermolecular bridges to stabilize the interface. (**C**) Protein–RNA interfacial modulation: natural products bind to composite protein–RNA interfaces, restricting local conformational flexibility and locking molecular switches.

Natural products modulate biomolecular interfaces not only in protein–protein interactions, but also in nucleic acid–nucleic acid and protein–nucleic acid interfaces [146], and can even act on intramolecular interfaces arising from nucleic-acid folding (Figure 3) [147,148]. In nucleic acid systems, natural products typically rely on the planarity of their π-conjugated systems or the shape complementarity of their macrocyclic scaffolds to localize to these nucleic acid-related interfaces through modes such as external end-stacking or intermolecular bridging. In this process, they can bridge RNA–RNA interfaces to suppress complex dissociation, bind protein–rRNA composite interfaces between ribosomal subunits to modulate functional states, and can even stabilize intramolecular interfaces of G-quadruplexes via stacking of their own π-conjugated scaffolds onto the terminal G-tetrad surface [148].

To elaborate the diverse mechanisms by which natural products act on nucleic acid-related interfaces, the following text will discuss six cases: G-quadruplex interfacial stabilizers, represented by daunomycin, berberine, and telomestatin; RNA–RNA interfacial bridging agents, represented by aminoglycosides; and inter-subunit ribosomal interface modulators, represented by tuberactinomycins and thiopeptide antibiotics.

#### 3.3.1. Daunomycin

Telomeric DNA is rich in GGGG repeat sequences, which spontaneously fold into G-quadruplex structures in the presence of potassium ions. This telomeric G-quadruplex interacts with various proteins and is associated with gene expression regulation. Multiple spectroscopic experiments, including circular dichroism (CD) and UV-Vis, demonstrated that the anthracycline natural product daunomycin can form specific binding with telomeric G-quadruplexes [64]. This binding mode is not a classical intercalation but involves an external binding mode comprising groove binding and end-stacking. The CD titration results showed only a slight red shift of approximately 1–3 nm. Furthermore, ^31^P NMR did not exhibit the characteristic downfield shift at around 1.6 ppm as typically observed in intercalation reactions. Differential scanning calorimetry results show daunomycin binding enhances the thermal stability of telomeric G-quadruplex DNA with an observed ΔTm~10 °C [64]. This thermal stabilization interferes with telomerase access to the functional site of telomeres, leading to telomere dysfunction [64]. Beyond this telomeric effect, daunorubicin also exhibits anticancer activity by stabilizing promoter G-quadruplexes and repressing WT1 transcription in leukemia cells [65].

#### 3.3.2. Berberine

Berberine, a natural alkaloid with an extended π-delocalized system, can also bind to and stabilize human telomeric G-quadruplexes, thereby suppressing cancer cell proliferation [66]. A combined multi-technique approach, including spectroscopic, calorimetric, and molecular modeling studies, demonstrated that berberine stabilizes the telomeric G-quadruplex complex through stacking interactions. Molecular modeling results indicate that berberine stacks onto the external G-quadruplex via π-π interactions. And it achieves stability through electrostatic interactions between the positively charged N7 nitrogen of berberine and the O6 carbonyl group of guanine. The relatively small enthalpy change (ΔH = −1.7 kcal/mol) coupled with a significant entropy increase contribution (TΔS = 6.5 kcal/mol) indicates that this entropy-driven thermodynamic behavior is consistent with end-stacking [67]. The heat capacity and hydration effects correlate with the burial of nonpolar and polar surface areas upon binding. Furthermore, CD experimental results indicate that while the 22-mer human telomeric sequence can adopt multiple interconvertible conformations (parallel/antiparallel/mixed-hybrid) under K^+^ conditions, in the presence of berberine (1:1), CD data reveal that berberine does not induce large-scale conformational rearrangement but selectively stabilizes the pre-existing mixed-hybrid G-quadruplex conformation from the K^+^-induced conformational ensemble [67,68].

#### 3.3.3. Telomestatin

Telomestatin is a natural product isolated from Streptomyces anulatus and reported to be an exceptionally potent telomerase inhibitor (IC_50_ = 5 nM) [69]. The structural similarity between telomestatin and the G-tetrad suggests that its inhibitory mechanism functions by promoting G-quadruplex formation or trapping preformed G-quadruplex structures. This sequesters the single-stranded d[T_2_AG_3_]_n_ primer molecule, which is required for telomerase activity. Telomestatin can promote or stabilize intramolecular G-quadruplex structures formed by d[T_2_AG_3_]_4_. However, under the same conditions, the mutated control sequence d[T_2_AGAG]_4_ does not undergo conversion. Simulated annealing docking studies suggest that telomestatin binds preferentially via external end-stacking onto the G-quartet surface, with a 2:1 (telomestatin/G-quadruplex) stoichiometry. This yields an overall binding energy of −191.6 kcal/mol, which is more favorable than the intercalative binding mode (−63.1 kcal/mol) [70]. Structurally, the electropositive center formed by the nitrogen atom in telomestatin interacts electrostatically with the electronegative carbonyl oxygen atom of guanine, while specific oxazole rings of telomestatin engage in hydrogen bonding and π–π stacking interactions with the quartet bases. This demonstrates how a macrocyclic scaffold constructs a multimodal interaction network across the entire quartet interface through shape complementarity with G-tetrad, thereby achieving both high affinity and structural selectivity [70]. It can impair telomerase-dependent telomere maintenance and promote senescence or apoptosis in cancer cells [71,72].

#### 3.3.4. Aminoglycosides

HIV-1 genomic RNA dimerization is mediated by the dimerization initiation site (DIS), a 5′-UTR stem-loop (SL1) whose self-complementary loop seeds formation of a kissing-loop dimer [73]. Perturbation of this element triggers system-level consequences, resulting in unstable and/or aberrant RNA dimers. As a result, RNA packaging and reverse transcription processes are impaired, causing a significant reduction in viral infectivity. Leveraging the A-site-like architecture of the loop-loop helix, structural analyses demonstrated that 4,5-disubstituted 2-deoxystreptamine (DOS) aminoglycosides such as neomycin, paromomycin and lividomycin can selectively engage the DIS interface. Crystal structures reveal two aminoglycosides bound per kissing-loop complex, with an average inter-ligand separation of 4.4 Å [73]. Each aminoglycoside acts by combining direct contact with water/cation-mediated interactions to engage with both strands of RNA, thereby forming intermolecular bridges at RNA–RNA junctions. In a radioactive RNA labeling competition experiment, radiolabeled homodimers were challenged with excess unlabeled heterologous RNA. In the absence of drug (or in the presence of neamine or ribostamycin), over half of the radiolabeled material is redistributed into heterodimers within 2 min. However, following treatment with the bridging DOS aminoglycosides, only minimal heterodimer accumulates after one hour, while the amount of homodimer remains nearly constant. The experimental results indicate that the action of the drug is to reduce the dissociation of already formed kissing-loop complexes, rather than to promote dimerization [73,74].

#### 3.3.5. Tuberactinomycins

As a class of anti-tuberculosis natural products, the tuberactinomycins viomycin and capreomycin inhibit protein synthesis by binding at the interface between the two ribosomal subunits. They are among the most effective options for combatting multidrug-resistant strains. In this study, the authors determined co-crystal structures of the Thermus thermophilus 70S ribosome programmed with a short mRNA and three full-length tRNAs in the classical A, P and E sites, and bound to either viomycin (3.3 Å) or capreomycin (3.5 Å) [75]. The two ligands both occupy space in the binding site located at the inter-subunit bridge B2a, which is not a cavity within a single subunit. Instead, it is an inter-subunit interface formed by the 16S rRNA helix h44 and the 23S rRNA helix H69 [75]. These drugs restrict the conformational freedom of three critical rRNA nucleotides (A1492, A1493, and A1913) by binding to this composite interface [76].

#### 3.3.6. Thiopeptides

Unlike many other antibiotics that bind active sites on the ribosome, thiopeptide antibiotics such as thiostrepton (Thio), nosiheptide (Nosi), and micrococcin (Micro) target the protein–rRNA interface, at the base of the stalk base (SB) of GTPase-associated center, thereby exerting antibacterial activity. The protein–rRNA interface in the ribosome is formed by the N-terminal domain of L11 (L11-NTD) and the H43 and H44 helices of 23S rRNA [77]. Structural data have shown that the Thio/Nosi binding site on the protein–rRNA interface contains a steric clash with the ribosomal positioning of EF-G domain V. Thio/Nosi locks two molecular switches on L11 by occupying this interface: the interdomain Switch 1 controlling cleft opening and closing, and the intradomain Switch 2 regulating the L11-L7 interaction interface. By locking these two molecular switches, Thio/Nosi alters the interface state near the SB and the interaction network among multiple components. Under standard EF-G binding conditions, the L11-NTD shifts to widen the cleft, placing it in Switch 1 on state. However, the tails of Thio/Nosi fix the L11-NTD in a closed conformation, preventing the SB cleft from opening. Simultaneously, through their interactions with H43/H44 and L11-NTD, these drugs lock Switch 1 in the off position. This prevents EF-G domain V from entering and stably binding to the stalk base [78]. Thus, Thio/Nosi not only physically competes with EF-G but also prevents its stable accommodation by restricting the conformational flexibility of L11. In addition, intradomain conformational changes within L11-NTD induced by Thio/Nosi result in a steric clash between the *N*-terminus of L11 and helix α4 of the L7-CTD. This disrupts the native structural complementarity within the interface of L11-L7, resulting in Switch 2 being locked off. Without the proper recruitment of the L7-CTD to L11, L7 cannot be correctly positioned onto the G’ subdomain of EF-G. All of these factors result in a decrease of inorganic phosphate (Pi) release and inhibition of multiple EF-G turnovers by Thio/Nosi [78].

Ultimately, thiopeptide natural products reshape the functional landscape of a critical protein–rRNA interface through differential occupation and conformational remodeling, thereby altering its multifunctional role in the translation elongation cycle. Interestingly, Micrococcin targets the same interface but maintains the on state of Switch 2, thus stabilizing rather than disrupting the L11-L7 interaction. The different functional consequences arising from thiopeptide-SB interface interactions are highlighted, which are linked to their fundamental property of enabling differential remodeling through structurally related ligands [78].

## 4. Time-Based Strategies

**Figure 4:**
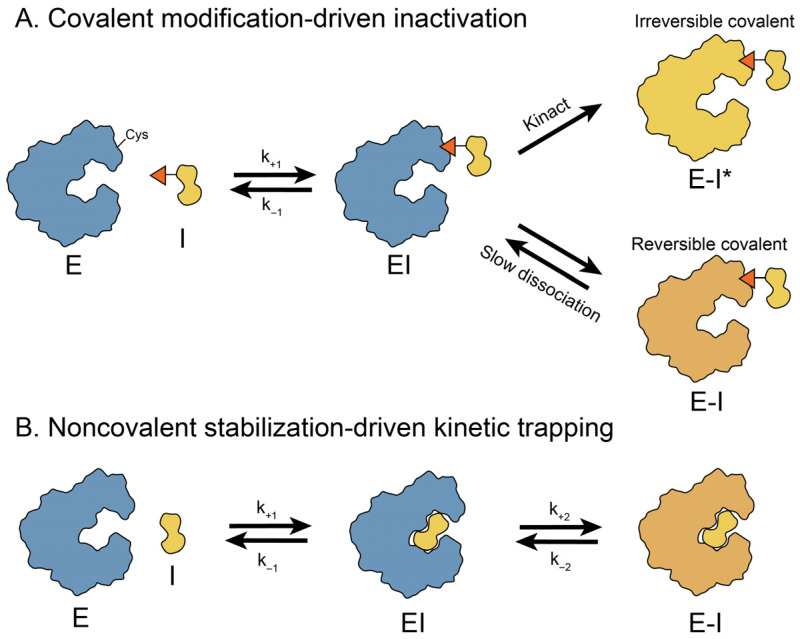
Representative modes of time-based action by natural products. (**A**) Covalent modification-driven inactivation: the natural product (I) first reversibly binds to the target protein (E) to form an initial noncovalent complex (EI), subsequently forming a covalent linkage to lock the target. This covalent engagement results in either kinetically irreversible inactivation (yielding the E-I* state) or a reversible covalent intermediate characterized by exceptionally slow dissociation (yielding the E-I state). (**B**) Noncovalent stabilization-driven kinetic trapping: rather than forming a covalent bond, the natural product functions through a noncovalent interaction network. After the initial complex (EI) is formed, the ligand acts by stabilizing transient conformational states or binding as a transition-state analogue, thereby inducing a slow-dissociating, kinetically trapped state (E-I).

Natural products regulate biological systems through more than mere occupation of spatial binding sites or remodeling of molecular interfaces. The key determinant of in vivo pharmacological activity and duration is not the binding affinity but, instead, the lifetime of the receptor-ligand complex, defined as the residence time [149]. By intervening in catalytic cycles or conformational transition pathways, natural products can selectively trap key intermediates, thereby introducing a temporal dimension to target regulation [150]. By prolonging the lifetimes of these intermediate states, they reshape downstream reactions and modulate biomacromolecular activity and turnover. In mechanistic terms, this time-based strategy can be broadly divided into two classes: covalent modification-driven inactivation and noncovalent stabilization-driven reversible inhibition. In the former, bond formation drives the target into a kinetically irreversible or quasi-irreversible state; in the latter, exceptionally slow dissociation or hindered conformational exchange prolongs the lifetime of a specific bound intermediate (Figure 4).

### 4.1. Covalent Modification-Driven Inactivation

In this mode, a natural product locks its target in an inactivated state via covalent modification. The formation of the covalent linkage is often kinetically irreversible. From a temporal perspective, kinetically irreversible covalent binding represents the extreme limit of residence time, where the dissociation rate approaches zero and the lifetime of the intermediate complex theoretically approaches infinity [151]. As a result, the target (such as the proteasome, cell-wall biosynthetic enzymes, or protein-processing enzymes) is driven into kinetic trapping that results in sustained target inactivation [152]. Representative structural exemplars include salinosporamide A-induced covalent acylation of Thr1O^γ^, the acyl-PBP intermediate formed by penicillin G, and the covalent adduct formed between fumagillin and MetAP-2 [81,84,86,151].

#### 4.1.1. Salinosporamide A

Salinosporamide A provides a representative example suggesting that some natural products commonly discussed under the space occupation view can be more naturally interpreted in terms of a time-based strategy. The 2.8 Å-resolution X-ray crystal structure shows that Salinosporamide A is covalently bound to Thr1O^γ^ via an ester linkage to the carbonyl derived from the β-lactone ring of the inhibitor. By this mechanism, the system first enters a covalent acyl-enzyme intermediate state [79]. Subsequently, C3-O undergoes an intramolecular cyclization with the C-2 side chain, thereby driving the system toward a temporally defined terminal state—namely, formation of a tetrahydrofuran ring [80]. Notably, in its final cyclic ether form, Salinosporamide A is irreversibly bound by virtue of the ‘C-3O barrier’ to penetration by water and a fully protonated and deactivated *N*-terminus that collectively prevent deacylation and account for irreversible inhibition [81]. Together, these features convert the initially formed covalent intermediate into a deacylation-resistant, kinetically trapped terminal state, thereby explaining the irreversible inhibition of the proteasome by Salinosporamide A. This prolonged target engagement may also contribute to its anticancer efficacy [79,81].

#### 4.1.2. Penicillin G

As a prototypical β-lactam antibiotic, penicillin G acts as a suicide substrate of penicillin-binding proteins (PBPs) [82]. The interaction begins with rapid, reversible formation of a noncovalent Michaelis complex, followed by nucleophilic attack by a serine on the β-lactam ring to yield a relatively stable covalent acyl-PBP intermediate. By trapping PBPs in this covalent state, β-lactams prevent completion of the catalytic cycle. The slow deacylation (e.g., k_3_~8 × 10^−6^ s^−1^ for typical susceptible PBPs) of this acyl-PBP intermediate dictates its prolonged residence time, thereby underpinning the antibacterial activity of penicillin G [83].

In contrast, crystallographic and kinetic analyses indicate that the broad β-lactam resistance of S. aureus PBP2a is not mainly due to impaired initial binding (K_d_) or accelerated acyl-enzyme breakdown (k_3_), but rather to inefficient formation of the acyl-PBP intermediate (reduced k_2_). In PBP2a, upon acylation, Cα, Cβ and Oγ from Ser 403 must move 1.1 Å, 1.4 Å and 1.8 Å, respectively, and strand β3 must twist together with a rearrangement at the helix α2 N-terminus to avoid steric clashes with the ligand [84]. These energetically costly rearrangements increase the barrier to acyl-PBP formation, lowering the acylation rates (k_2_~0.22 s^−1^) and thereby conferring broad-spectrum β-lactam resistance [84].

#### 4.1.3. Fumagillin

Fumagillin achieves kinetic capture in the time dimension through irreversible covalent modification. Based on the 1.8 Å crystal structure together with the difference electron-density maps, the epoxide of the natural product fumagillin forms a C-N covalent bond with His231 in the active site of MetAP-2 [85]. This covalent chemistry effectively locks the enzyme in a kinetically trapped state, thereby leading to a sustained inhibitory effect on angiogenesis-related signaling [86,87]. Further, structural comparison of Type 1 and Type 2 MetAP inhibitor complexes indicates that the Type 1 enzyme has a more sterically restricted active-site cavity. Consequently, accommodating the ovalicin/fumagillin scaffold requires modest displacement of active-site residues and a pronounced~134° rotation of the His310 side chain to avoid steric conflict. This steric constraint provides a structural rationale for the much weaker binding of ovalicin/fumagillin for MetAP-1 over MetAP-2 [85].

### 4.2. Noncovalent Stabilization-Driven Reversible Inhibition

By contrast, another class of natural products does not fully lock the target. Instead, these molecules act through noncovalent interactions [152]. They freeze the short-lived conformational states or bind as transition-state analogues, thereby markedly extending the lifetime of these states [153]. This time-based stabilization delays the rearrangements needed to reach catalytically competent states and slows the associated chemical steps [154]. Illustrative cases include pentostatin as a transition-state analogue of adenosine deaminase (ADA), ouabain stabilizing the E2P state of Na^+^/K^+^-ATPase, and cytochalasin B binding the inward-open conformation of human GLUT1 [89,93,95].

#### 4.2.1. Pentostatin

Adenosine deaminase (ADA) is proposed to catalyze hydrolytic deamination via initial OH^-^ addition to the substrate C6, generating a tetrahedral intermediate, which subsequently collapses with elimination of NH_3_ to yield the product. Pentostatin is a highly potent transition-state analogue of ADA, the tetrahedral carbon at its C8 position mimics the tetrahedral transition-state intermediate [88]. It binds ADA through an extensive noncovalent network and is clinically used to enhance the efficacy of several antitumor and antiviral adenosine analogues [89,90]. In the 2.6 Å ADA–DCF complex, the 8R-hydroxyl (O8) coordinates the Zn^2+^ cofactor, anchoring the inhibitor in the deep active-site pocket. In parallel, additional interactions with residues such as His17, Asp19, and Glu217 further stabilize the bound complex [89]. Kinetically, pentostatin behaves as an exceptionally tight-binding, slow-dissociating ADA inhibitor: k_on_ ≈ 2.6 × 10^6^ M^−1^s^−1^ and k_off_ ≈ 6.6 × 10^−6^ s^−1^, giving a dissociation half-life of ~29 h [89,91].

#### 4.2.2. Ouabain

In the space and interface dimensions, ouabain binds the extracellular CTS binding pocket formed by transmembrane segments αM1-6 [92]. In the high-affinity [Mg]E2P-ouabain complex, αM4 winds up by one helix turn, extending the ouabain-binding cavity toward cation site II, which allows ouabain to move ~2 Å deeper into the cavity [93]. In the time dimension, this structural progression selectively conformationally traps the E2P intermediate of Na^+^,K^+^-ATPase, substantially prolonging the lifetime of this transient state. Through this stabilization, ouabain effectively acts as a kinetic trap, thereby blocking conformational transitions and slowing its dephosphorylation kinetics. Quantitatively, dephosphorylation kinetics show that ouabain stabilizes the E2P phosphoenzyme: fitting the reported time courses to an apparent first-order decay gives k_dephos_~0.046 s^−1^ (t_1/2_~15 s) for E2P, but k_dephos_~0.014 s^−1^ (t_1/2_~50 s) for the E2P-ouabain complex [93]. Functionally, this ouabain-bound inhibitory state inhibits tumor cell proliferation and induces apoptosis. These effects have been linked to the NKA α3 isoform [94].

#### 4.2.3. Cytochalasin B

Cancerous cells have an acutely increased demand for energy, leading to increased levels of human glucose transporter 1 (hGLUT1). This has motivated interest in hGLUT1 as a potential inhibitor target. By solving the crystal structure of full-length WT-hGLUT1 with the natural product cytochalasin B, the authors show that the inhibitor binds within the transporter’s central cavity in an inward-open conformation [95,96]. Furthermore, the binding of compound GLUT-i1 reveals a shared inhibitory mechanism. This binding restricts the movement of Trp388 and prevents the helix TM10 from entering the central cavity, thereby impeding the alternating-access cycle. Mechanistically, cytochalasin B prolongs the residence time of the inward-open state and blocks conversion to the outward-facing state through kinetic trapping [95]. Functionally, cytochalasin B inhibits hGLUT1-mediated glucose transport with submicromolar potency. Competitive assays confirm that its inhibitory potency decreases as glucose concentration increases, with the IC_50_ value rising from 0.11 µM at 0.02 mM glucose to 2.2 µM at 1 mM glucose, consistent with direct competition at the central substrate-binding site [95].

## 5. Conceptual Boundaries and Practical Utility of the SIT Framework

### 5.1. Conceptual Position and Boundaries of the SIT Framework

As shown in Table 1, the SIT framework is not intended to replace traditional target-, phenotype-, or structure-based classifications, particularly because these established systems remain useful for organizing natural products from distinct pharmacological or chemical perspectives [155,156,157]. Rather, SIT serves as a complementary framework by focusing on how natural products reshape biomolecular function through spatial occupancy, interfacial remodeling, and regulation of state lifetimes. In this sense, SIT adds a unified interpretive layer for comparing mechanisms across biomolecular systems.

A further value of the SIT framework lies in its ability to position mechanistic concepts operating at different levels within a unified interpretive system, thereby providing a more structured and comparable explanation of natural product action. For example, molecular glues are best understood as an important mode within the Interface dimension, rather than as an alternative to SIT. Similarly, allostery is more appropriately regarded as a functional outcome [158], since it may arise from spatial wedging, interfacial remodeling, or the cooperation of both. Induced fit and conformational selection should be treated as models of binding or molecular recognition [159], rather than as classification schemes for natural product mechanisms. By organizing such concepts within a common framework, SIT can facilitate both the analysis and design of complex, multidimensional mechanisms of action.

The SIT framework has three main limitations. Firstly, its application generally requires detailed mechanistic evidence and often involves coupled effects across dimensions. In many natural-product systems, although the three dimensions are analytically distinguishable, they are often mechanistically coupled and may occur sequentially or simultaneously at different stages of action. Secondly, current mechanistic interpretation still relies heavily on static structural snapshots, which often fail to capture low-population or short-lived states such as cryptic pockets. Thirdly, even when SIT-relevant features can be defined in vitro, linking such mechanistic assignments to in vivo pharmacology remains challenging. SIT should therefore be viewed as a complementary framework with high explanatory power, rather than as a universal replacement for classical classification systems.

In addition, the evidence base across the three SIT dimensions is currently asymmetric. Studies related to the Space dimension are the most well-established, reflecting the fact that natural product research has long accumulated extensive structural and biochemical evidence under the classical paradigm of binding-site occupancy. By comparison, research on the Interface dimension has expanded more recently, largely with the rise of concepts such as molecular glues and interface stabilization. Evidence for the Time dimension remains the most limited, in part because state lifetimes and transient intermediates are intrinsically more difficult to capture directly, and because the literature has emphasized static structures and endpoint affinity rather than kinetic processes. Accordingly, the uneven density of examples across the three dimensions in this review reflects the present state of the field and the availability of evidence, rather than any subjective prioritization by the authors. If anything, the relative underdevelopment of the time dimension further highlights it as one of the most promising frontiers in future studies of natural product mechanisms [9,160].

### 5.2. Practical Utility of the SIT Framework

Although traditional classification schemes can define the chemical or pharmacological category to which a natural product belongs, they are often less effective at extracting the mechanistically decisive variables that should guide subsequent optimization. For example, target-based classification can easily equate action on the same target with a shared optimization logic, whereas phenotype-based classification may obscure mechanistic differences underlying similar functional outputs [161]. By contrast, the SIT framework links optimization priorities directly to the dominant mechanism. In the space dimension, the key issues are geometric complementarity, site occupancy, and conformational restriction within active sites or grooves. In the interface dimension, priority shifts to multicomponent complex assembly, bridging, and stabilization. In the time dimension, the central variables are the lifetime and kinetic stabilization of reactive intermediates or functional states. Thus, the importance of SIT lies not only in reorganizing natural product mechanisms but also in converting mechanistic judgment into a more explicit optimization strategy.

This advantage becomes especially clear in concrete case studies. Rapamycin shows that optimization should not be limited to strengthening binding to an individual protein [47], but should instead focus on the FKBP12–rapamycin–FRB ternary complex as the active entity [48]. In the space dimension, scaffold-based systems such as AgRP and Kalata B1 indicate that conformational preorganization should be prioritized as an optimization objective [42]. The stictic acid–p53 system shows that, when supported by convergent multidimensional evidence, optimization can be directed more precisely toward cryptic-pocket engagement and conformational rescue [28]. In the interface dimension, brefeldin A suggests that the truly druggable species may be a transient complex itself rather than any isolated component, whereas fusicoccin A demonstrates that interfacial stabilization can be translated into quantifiable optimization goals, such as enhanced complex affinity and stability [15,63]. In the time dimension, ouabain illustrates that the most informative variable to track may not be static binding strength, but rather the lifetime and kinetic prolongation of a specific functional state [93]. Taken together, these examples show that the SIT framework is useful not only for explaining how natural products work, but also for defining what should be optimized, measured, and tracked in subsequent mechanistic and translational studies.

## 6. Outlook and Perspectives

Although the ‘Space–Interface–Time’ framework articulated in this review provides a systematic perspective for understanding the functional logic of natural products, its application to the regulation and stabilization of biomolecular conformations still faces multiple challenges spanning theoretical understanding, technical characterization, and therapeutic translation.

At present, our understanding of natural product-target interactions is built primarily upon static structural snapshots obtained from methods such as X-ray crystallography and conventional cryo-EM [162,163]. However, they often struggle to capture low-population, transient states that are tightly coupled to function, including cryptic pockets, transient ternary complexes, and short-lived intermediates [164,165]. This incomplete view of the dynamic conformational landscape impedes the deep exploration of novel druggable sites, particularly for conventionally “undruggable” oncogenic drivers [102,164,166]. Much of the field still relies on simplified in vitro models. Conventional cell-free biophysical assays (e.g., SPR and ITC) offer the advantage of controlled experimental conditions [167,168], but they struggle to replicate the complex physiological environment within cells, such as macromolecular crowding, liquid–liquid phase separation, and intricate networks of post-translational protein modifications, which are often dysregulated in cancer cells [169,170,171]. As a result, the affinity or potency metrics obtained in vitro assays typically cannot be linearly extrapolated to in vivo therapeutic outcomes [10,172,173]. These data often overlook the complex physiological barriers in the body (such as metabolism, plasma protein binding and tumor microenvironment) as well as dynamic in vivo pharmacokinetic profiles [10,174]. This severe disconnect between molecular-level mechanisms of action and systemic pharmacokinetics has become a major bottleneck in the therapeutic translation of natural product-derived drugs.

Future research should move beyond static structural snapshots toward dynamic analysis of conformational transitions [165,175]. The emergence of cutting-edge technologies such as time-resolved cryo-EM and time-resolved serial femtosecond crystallography has endowed structural biology with unprecedented temporal resolution. This enables us to capture conformational changes in targets and non-equilibrium intermediate states of complexes on timescales ranging from milliseconds to femtoseconds [165,176]. Leveraging the growth of kinetic data, AI-assisted computational methods—such as AlphaFold, PocketMiner, and molecular dynamics (MD) simulations—can help nominate cryptic pockets and alternative target states for experimental testing, offering new avenues for conquering traditionally “undruggable” tumor-driving targets [102,164,166].

By systematically integrating emerging technologies, we can not only discover new cryptic pockets for drug binding but also combine potent natural products with targeted ligands such as antibodies and aptamers or nano-delivery technologies. This approach enables us to overcome the challenges posed by the complex tumor microenvironment and achieve the precise delivery and release of these potent natural products. Ultimately, by leveraging a closed-loop validation strategy based on kinetic parameters, it is possible to link time-resolved structural dynamics information with in vivo target occupancy, residence time, and pharmacokinetic/pharmacodynamic (PK/PD) parameters. This enables the development of a translational model that bridges the gap between in vitro mechanisms and in vivo antitumor efficacy.

## Figures and Tables

**Table 1:** Comparison of the SIT framework with classical paradigms.

Paradigm	Organizing Principle	Strengths	Limitations
Target-based	Grouped by biomolecular targets	Intuitive and compatible with current drug discovery workflows	Prioritizes the target over the mechanism and polypharmacology
Phenotype-based	Grouped by dominant phenotypic outcomes	Useful when targets are unknown and can link phenotypic screening to disease	Obscures mechanistic differences behind similar phenotypes
Structure-based	Grouped by chemical scaffolds or biosynthetic origin	Valuable for scaffold organization, SAR analysis, and structural optimization.	Fails to reliably predict mechanism or function from structural similarity
SIT Framework	Grouped by spatial, interfacial, and temporal modes of action	Effective for cross-system comparison and mechanistic analysis beyond specific structures or diseases	Requires detailed evidence and may involve coupled effects across dimensions

**Table 2:** Representative natural products under the SIT framework.

Dimension	Natural Product	Biomolecular System	Key Evidence	Mechanistic Outcome	Refs.
Space	Paclitaxel	Protein (tubulin/microtubule)	structural analysis (X-ray/cryo-EM), mutagenesis	taxane pocket occupancy, microtubule stabilization	[17,18,19,20]
	(+)-Discodermolide	Protein (tubulin/microtubule)	structural analysis (X-ray/NMR), binding analysis (PAL/radioligand competition), dynamics analysis (HDX-MS)	taxane pocket occupancy, microtubule stabilization	[21,22]
	Staurosporine	Protein (Lck)	structural analysis (X-ray), binding analysis (SPR), mutagenesis	ATP-binding site occupancy, Lck kinase inhibition	[23,24,25]
	Geldanamycin	Protein (Hsp90)	structural analysis (X-ray), binding analysis (HPLC), mutagenesis	ATP-binding site occupancy, Hsp90 refolding disruption, client protein degradation	[26,27]
	Stictic acid	Protein (p53)	dynamics analysis (MD), binding analysis (DSF), mutagenesis, functional assays (luciferase reporter)	L1/S3 pocket occupancy, Wild-type-like conformational refolding, p53 reactivation	[28,29]
	Netropsin	Nucleic acid (minor groove of A/T-rich DNA)	structural analysis (X-ray)	minor-groove binding, blockade of DNA unwinding	[30,31,32]
	SCPCHD	Nucleic acid (minor groove of A/T-rich DNA)	structural analysis (X-ray/NMR/fiber-diffraction/CD)	minor-groove binding, blockade of DNA unwinding	[33,34]
	Mithramycin	Nucleic acid (minor groove of G/C-rich DNA)	structural analysis (X-ray/NMR), binding analysis (FP)	minor-groove binding, EWS–FLI1 transcriptional inhibition	[35,36]
	Paromomycin	Nucleic acid (decoding A site in ribosome)	structural analysis (X-ray)	16S rRNA A-site occupancy, induction of a decoding-like switch, translational misreading	[37,38]
	Tigecycline	Nucleic acid (tetracycline-binding site in ribosome)	structural analysis (X-ray), binding analysis (radioligand competition), dynamics analysis (smFRET), functional assays (IVTT)	16S rRNA binding, disruption of tRNA recognition, steric evasion of TetM rescue	[39,40]
	Kalata B1	Protein (VEGF-R2)	structural analysis (RP-HPLC/NMR), functional assays (MTS), stability assay (serum)	epitope stabilization, VEGF-A antagonism	[41,42,43]
	MCoTI-I	Protein (Hdm2/HdmX)	structural analysis (NMR), binding analysis (FP/FRET/Co-IP), functional assays (IHC)	epitope stabilization, Hdm2/HdmX pocket binding, Hdm2/HdmX antagonism	[44]
	AgRP	Protein (αvβ3 integrin)	computational analysis (modeling), functional assays (yeast-display/FACS/cell adhesion)	epitope stabilization, αvβ3 integrin antagonism	[45,46]
Interface	Rapamycin	Protein (FKBP12-mTOR FRB interface)	structural analysis (X-ray/SIRAS), binding analysis (SPR)	FKBP12–rapamycin–FRB stabilization, mTORC1 inhibition	[47,48,49]
	IAA	Protein (TIR1-Aux/IAA interface)	structural analysis (X-ray), binding analysis (radioligand competition/SPR), mutagenesis	TIR1–Aux/IAA stabilization, Aux/IAA degradation	[50,51,52]
	JA-Ile	Protein (COI1–JAZ interface)	structural analysis (X-ray), binding analysis (radioligand competition), mutagenesis, functional assays (Y2H)	Co-receptor surface stabilization, JAZ degradation,	[53,54,55]
	Sanglifehrin A	Protein (CYPA-KRAS G12C interface)	structural analysis (X-ray/native PAGE), dynamics analysis (TR-FRET/split-luc), mutagenesis	CYPA/RMC-4998/KRAS G12C assembly, CYPA-mediated steric blockade, KRAS pathway inhibition	[56,57]
	Resveratrol	Protein (transthyretin dimer-dimer interface)	structural analysis (X-ray), binding analysis (ITC), functional assays (ThT/Turbidity)	tetrameric interface stabilization, subunit dissociation blockade, inhibition of amyloid fibrillation	[58,59]
	Brefeldin A	Protein (ARF1•GDP–Sec7 interface)	structural analysis (X-ray), computational analysis (APBS), binding analysis (FA), mutagenesis	ARF1•GDP–Sec7-BFA stabilization, GEF inhibition/ARF1 blockade	[15,60,61]
	Fusicoccin A	Protein (14-3-3-PM H^+^-ATPase interface)	structural analysis (X-ray), computational analysis (docking), binding analysis (ITC/FP/Co-IP)	14-3-3-PM H^+^-ATPase stabilization, H^+^-ATPase activation	[62,63]
	Daunomycin	Nucleic acid (terminal G-quartet surface)	structural analysis (NMR/UV-Vis/FL/CD), computational analysis (docking), binding analysis (SPR), stability assay (DSC)	G-quadruplex stabilization, telomerase interference, WT1 repression	[64,65]
	Berberine	Nucleic acid (terminal G-quartet surface)	conformational analysis (UV-Vis/FL/CD), binding analysis (ITC/osmotic stress), computational analysis (docking)	selective G-quadruplex stabilization	[66,67,68]
	Telomestatin	Nucleic acid (terminal G-quartet surface)	structural analysis (native PAGE/mutant control), computational analysis (docking)	G-quadruplex stabilization, primer sequestration, telomerase inhibition	[69,70,71,72]
	Aminoglycosides	Nucleic acid (HIV-1 DIS RNA interface)	structural analysis (X-ray), binding analysis (ITC/footprinting), stability assay (UV-melting)	DIS kissing-loop stabilization, impair RNA packaging and reverse transcription	[73,74]
	Tuberactinomycins	Nucleic acid (16S–23S rRNA interface)	structural analysis (X-ray/cryo-EM), dynamics analysis (MD), comparative structural analysis	inter-subunit interface stabilization, pretranslocation-state stabilization, protein synthesis inhibition	[75,76]
	Thiopeptides	RNA–protein complex (23S rRNA-L11 interface)	structural analysis (X-ray), binding analysis (footprinting/SDS-PAGE), mutagenesis, functional assays (GTP hydrolysis), comparative structural analysis	L11-rRNA interface stabilization, EF-G turnover inhibition, translation elongation blockade	[77,78]
Time	Salinosporamide A	Protein (20S proteasome β5 peptidase)	structural analysis (X-ray), dynamics analysis (MD/QM/MM), comparative structural analysis	deacylation-resistant kinetic trapping, irreversible proteasome inhibition	[79,80,81]
	Penicillin G	Protein (PBP transpeptidase)	structural analysis (X-ray), dynamics analysis (RQF), comparative structural analysis	acyl-PBP intermediate trapping, irreversible PBP inhibition	[82,83,84]
	Fumagillin	Protein (MetAP2)	structural analysis (X-ray), comparative structural analysis	covalent C-N bond formation irreversible MetAP-2 inhibition	[85,86,87]
	Pentostatin	Protein (ADA)	structural analysis (X-ray), dynamics analysis (k_on_/k_off_) comparative structural analysis	transition-state mimicry, long-residence ADA inhibition	[88,89,90,91]
	Ouabain	Protein (Na^+^,K^+^-ATPase)	structural analysis (X-ray/cryo-EM), dynamics analysis (k_on_/k_off_)	E2P intermediate trapping, slowed dephosphorylation, Na^+^,K^+^-ATPase inhibition	[92,93,94]
	Cytochalasin B	Protein (GLUT1)	structural analysis (X-ray), computational analysis (docking), mutagenesis, functional assays (inhibition/glucose competition)	inward-open state trapping, alternating-access cycle blockade, Glucose transport inhibition	[95,96]

## Data Availability

No new data were created or analyzed in this study. Data sharing is not applicable to this article.

## Abbreviations

The following abbreviations are used in this manuscript:

APBS	Adaptive Poisson–Boltzmann solver
CD	Circular dichroism
Co-IP	Co-immunoprecipitation
cryo-EM	Cryo-electron microscopy
DSC	Differential scanning calorimetry
DSF	Differential scanning fluorimetry
FA	Fluorescence anisotropy
FL	Fluorescence spectroscopy
FP	Fluorescence polarization
FRET	Fluorescence resonance energy transfer
Hdm2/HdmX	Human double minute 2/Human double minute X
HDX-MS	Hydrogen–deuterium exchange mass spectrometry
HPLC	High-performance liquid chromatography
IHC	Immunohistochemistry
ITC	Isothermal titration calorimetry
IVTT	In vitro transcription/translation
k_on_/k_off_	Association rate constant/Dissociation rate constant
KRAS G12C	A mutant form of KRAS in which glycine at position 12 is replaced by cysteine
MD	Molecular dynamics
MTS	MTS cell viability assay
native PAGE	Native polyacrylamide gel electrophoresis
NMR	Nuclear magnetic resonance
PAL	Photoaffinity labeling
QM/MM	Quantum mechanics/Molecular mechanics
RGD	Arg-Gly-Asp
RP-HPLC	Reversed-Phase high-performance liquid chromatography
RQF	Rapid-Quench Flow
SAR	Structure-Activity Relationship
SDS-PAGE	Sodium dodecyl sulfate polyacrylamide gel electrophoresis
SIRAS	Single isomorphous replacement with anomalous scattering
smFRET	Single-molecule FRET
split-luc	Split luciferase assay
SPR	Surface plasmon resonance
ThT	Thioflavin T fluorescence assay
TR-FRET	Time-resolved FRET
UV-Vis	Ultraviolet-visible spectroscopy
X-ray	X-ray crystallography
Y2H	Yeast two-hybrid
